# A Voxel-Based Morphometric Study of Gray Matter in Specific Phobia

**DOI:** 10.3390/life13010119

**Published:** 2022-12-31

**Authors:** Francisco Rivero, Rosario J. Marrero, Teresa Olivares, Wenceslao Peñate, Yolanda Álvarez-Pérez, Juan Manuel Bethencourt, Ascensión Fumero

**Affiliations:** 1 Departamento de Psicología Clínica, Psicobiología y Metodología, Facultad de Psicología, Universidad de La Laguna, 38200 La Laguna, Tenerife, Spain; 2 Departamento de Psicología, Facultad de Ciencias de la Salud, Universidad Europea de Canarias, 38300 La Orotava, Tenerife, Spain; 3 Instituto Universitario de Neurociencia (IUNE), Universidad de La Laguna, 38200 La Laguna, Tenerife, Spain; 4 Fundación Canaria Instituto de Investigación Sanitaria de Canarias (FIISC), 38109 El Rosario, Tenerife, Spain

**Keywords:** MRI, prefrontal areas, putamen, gray matter volume, specific phobia

## Abstract

The objective of this study was to analyze the neurostructural abnormalities of brain areas responsible for the acquisition and maintenance of fear in small animal phobia by comparing gray matter volume (GMV) in individuals with phobia and non-fearful controls. Structural magnetic resonance imaging was obtained from 62 adults (79% female) assigned to one of two groups: 31 were diagnosed with small animal phobia and 31 were non-fearful controls. To investigate structural alterations, a whole-brain voxel-based morphometry analysis was conducted to compare the GMV of the brain areas involved in fear between both groups. The results indicated that individuals with a small animal specific phobia showed smaller GMV in cortical regions, such as the orbitofrontal (OFC) and medial frontal cortex, and greater GMV in the putamen than non-fearful controls. These brain areas are responsible for avoidant behavior (putamen) and emotional regulation processes or inhibitory control (prefrontal cortex (PFC)), which might suggest a greater vulnerability of phobic individuals to acquiring non-adaptive conditioned responses and emotional dysregulation. The findings provide preliminary support for the involvement of structural deficits in OFC and medial frontal cortex in phobia, contributing to clarify the neurobiological substrates for phobias.

## 1. Introduction

Anxiety disorders (ADs) have been shown to be one of the mental disorders with the highest prevalence rate [1]. Different types of ADs include generalized anxiety disorder (GAD), panic disorder, agoraphobia, social anxiety disorder (social phobia), specific phobias (SP), obsessive-compulsive disorder (OCT), and post-traumatic stress disorder (PTSD) [2]. These disorders share characteristics of excessive, disproportionate, and persistent worries and fears that interfere with activities of daily living. Biological, psychological, or cultural approaches have proposed different models in an attempt to identify the nature and conditions in which these disorders develop [2,3,4]. The biological approach suggests that particular genes and deficits of some neurotransmitters, such as serotonin, could be the cause of certain emotional disorders [5,6]. Accordingly, some people show greater vulnerability to developing ADs [7]. The psychological approach emphasizes fear learning processes associated with threat detection and defensive responses [3]. In this sense, theories about Pavlovian conditioning have had the greatest explanatory power [8].

Neuroimaging techniques have made it possible to identify the neurobiological substrates underlying ADs [9]. The neural basis of fear, in particular, has been extensively studied [10,11]. Fear not only implies a defensive response to a threatening stimulus but also a subjective experience of fear [8]. Fear neural systems are associated with the functional interaction of subcortical (amygdala, thalamus, and basal ganglia) and cortical (insular cortex, anterior cingulate cortex (ACC), OFC, and PFC) regions [12]. In functional studies, the left amygdala and insula, as well as the left dorsolateral PFC, left ACC, and fusiform gyrus, have shown greater brain activity in individuals with phobia, suggesting a double processing of phobic stimuli: a rapid emotional processing pathway through the limbic areas and a slow pathway involving an interaction of the limbic (emotional) and frontal (rational) areas [11]. Hence, imbalanced limbic-prefrontal processing is associated with a failure to regulate emotions, which may, in turn, contribute to maintaining fear [13]. Studies on transcranial magnetic stimulation have shown that fear lessens when the dorsolateral PFC is stimulated [14]. The stimulation of this area seems to prevent the reconsolidation of fear memory. However, the pathophysiology of ADs remains an ongoing debate, since the processes underlying anxiety and fear-related disorders are more complex. In fact, common and specific factors have been identified with the various diagnoses related to anxiety to the extent that different features of the phobic stimulus activate different brain areas. Thus, stimuli presenting near activated areas were associated with a motor response, whereas a greater number of phobic stimuli activated sensory areas [15], suggesting a different emotion regulation strategy.

Therefore, the main focus of this study was to deepen current understanding of neuromorphometric abnormalities in participants with SP. Voxel-based morphometry (VBM) studies provide additional information to functional studies on the emergence and maintenance of ADs; in other words, identifying neuroanatomical abnormalities of brain development could explain the pathologic condition [16]. The procedure mainly applied is gray matter volume (GMV) analysis. Thus, alterations in brain structures associated with dysfunctions in the processing and regulation of emotion, as well as fear conditioning processes, can be explored. In this regard, contradictory results have been found for both larger and smaller GMV in the areas traditionally linked to ADs [17,18,19,20]. Smaller subcortical and larger cortical GMVs have been associated with social anxiety disorder (SAD) compared with healthy controls [21,22]. In particular, reductions in GMV in the right thalamus, the left parahippocampus, and the bilateral putamen were found in SAD [22,23]. A reverse pattern of larger subcortical and smaller cortical GMVs has also been reported [24]. Specifically, an increased right putamen volume, and decreased lateral/medial PFC and left insula volumes have been found in generalized anxiety disorder (GAD) compared with fear-related AD [19]. Furthermore, smaller volume and altered activity patterns of the ventromedial PFC have been observed in patients with ADs [25]. On the other hand, a different pattern was revealed of both smaller cortical, such as PFC and temporal-parietal cortices, and subcortical GMVs, such as the striatum, thalamus and brain stem, in anxiety and panic disorder versus healthy controls [20,26]. The larger volume of cortical areas has been interpreted as a result of greater efforts to regulate emotions in individuals with AD. The smaller volume of subcortical areas, however, has been related to greater sensitivity to emotional stimuli and sustained emotional dysregulation that may lead to the progressive impairment of these areas [22]. Conversely, a smaller volume of cortical areas has been interpreted as deficits in top-down control [19], and a larger GMV of subcortical areas, such as the putamen, could facilitate the saliency of stimuli and be associated with fear conditioning [27].

Despite the findings on the structural alterations underlying ADs, the data are inconclusive, and studies on SPs are scant. To our knowledge, few morphometric studies on SPs have been reported. Spider-phobic individuals show smaller left amygdala volume than healthy controls [28]. In addition, the dorsomedial and dorsolateral PFC volume of individuals with dental phobia is smaller than that of non-phobic controls [29]. However, Hilbert et al. [30] found increased GMVs in left medial OFC, right subgenual ACC, and areas in the occipital cortex and cerebellum in individuals with dental and snake phobia compared with non-phobic controls. The greatest structural differences appeared between the group with dental phobia and the control group, mainly with increased GMV in OFC, dorsomedial PFC, subgenual ACC, occipital cortex, as well as the insula. Therefore, it seems that dental phobia requires more cognitive anxiety and fear processing compared to animal phobias. By contrast, in another study no statistically significant differences in GMV were found between individuals with dental phobia and healthy controls [31].

In the light of these findings, a significant heterogeneity for the brain structures involved in several ADs has been noted across studies. Moreover, the dearth of studies in SPs and the reduced number of participants highlight the need for further exploration of the VBM of the brain areas involved in SPs. The main aim of this study was to analyze whether the GMV of brain areas responsible for the acquisition and maintenance of fear was different between individuals with an SP of small animals (cockroaches, spider, mouses, and lizards) compared with non-fearful controls. The GMV of brain areas could be a cue of greater vulnerability to anxiety and fear-related disorders.

## 2. Materials and Methods

### 2.1. Participants

Participants included in the sample were 62 adults (21% male and 79% female) resident in Tenerife (Canary Islands, Spain) who were assigned to one of two groups: 31 were diagnosed with small animal phobia (19.4% males, ranging in age from 19 to 56 years, mean age = 35.16 years, SD = 11.10) and 31 were non-fearful controls (22.6% males, ranging in age from 18 to 41 years, mean age = 22.00 years, SD = 5.09).

### 2.2. Instruments

The Composite International Diagnostic Interview (CIDI), Version 2.1 [32], was used to test the diagnosis of phobia, and questions were asked about an SP, agoraphobia, social phobia, and panic attacks. In addition, participants’ sociodemographic features (sex and age) were assessed.

The S–R (Situation–Response) Inventory of Anxiousness [33] was administered to participants of both groups with and without phobia. This inventory was composed of 14 items with a 5-point Likert-type scale that assessed the most frequent symptoms (i.e., physiological, cognitive, and behavioral) associated with the response to an anxiogenic stimulus (i.e., cockroaches, spiders, lizards or mice). The inventory showed high internal consistency (0.95) and adequate convergent validity [34].

The Edinburgh Handedness Inventory [35] was used to determine that all participants were right-handed.

### 2.3. Design

A cross-sectional quasi-experimental design was carried out to assess the GMV of brain areas responsible for the acquisition and maintenance of fear in small animal phobia in individuals with phobia and non-fearful controls. A whole-brain voxel-wise analysis was performed to identify the brain regions in which there were structural differences between both groups.

All participants were right-handed, and none had any visual problems. The sample with phobia fulfills the following inclusion criteria: participants showed no impediment to undergoing a magnetic resonance imaging (MRI) session; all were adults with a diagnosis of SP, according to the scores in questionnaires on SP and anxiety and through a clinical interview, and were receiving no treatment for SP at the time of the study; the phobia had to be the primary psychological disorder that could not be explained by another health condition. The inclusion criteria for sample non-fearful controls were the same as for the phobia group, except that their score in the assessment questionnaire on SP was low.

### 2.4. Procedure

Participants with phobia were recruited from April to July 2018 through advertisements in the press, on websites, with flyers, and on TV. Concurrently, the control group was made up of psychology students who voluntarily participated in the research, which granted them additional credit in the subjects taught by the researchers. The procedure was explained to participants before they gave their informed consent. In morphometric experimental studies with ADs, there is some variability in terms of sample size. In a meta-analysis study in which 24 papers were identified, only four slightly exceeded 30 participants per group [24]. Particularly in the case of SPs, the sample size is usually below the 30 subjects for each experimental and control group [30,31]. In this study, the effect size was calculated through r^2^ for each of the differences found between the groups to identify the power of the study. This study adhered to the ethical standards of the Declaration of Helsinki and was approved by the Ethics Committee for Research and Animal Welfare of the University of La Laguna (CEIBA2013-0086).

### 2.5. MRI Data Acquisition

Whole-brain structural and functional MRI images were acquired on a 3.0 T MR scanner (GE 3.0T Sigma Excite HD) with a 12-channel head coil. During the scans, the subjects were instructed to keep their eyes closed, to relax but not to sleep, and to lie as still as possible. High-resolution three-dimensional T1-weighted images were acquired: repetition time (TR)/echo time (TE) = 8852 ms/1756 ms, flip angle = 10°, 172 sagittal slices, slice thickness = 1 mm, field of view (FOV) = 256 × 256 mm^2^, data matrix = 256 × 256 × 172, the voxel size was 1 × 1 × 1 mm and TI = 650 ms. Each scan was inspected by an experienced neuroradiologist to rule out visible movement artifacts and gross structural abnormalities before image processing.

### 2.6. MRI Processing

Processing of structural images was performed using the Computational Anatomy Toolbox for Statistical Parametric Mapping software (CAT12 for SPM12; http://www.fil.ion.ucl.ac.uk/spm/ (accessed on 1 September 2022)) [36]. First, all MRI images were manually reoriented on the anterior posterior commissure line for better registration. Second, the high-resolutionT1-weighted images were segmented into gray matter (GM), white matter and cerebral spinal fluid in CAT12. The GMV were analyzed using VBM. T2 images were obtained at the end of the protocol, in the event of a clinical finding appearing by chance. Third, registered images were transformed to the MNI space using the FORWARD strategy that allows for data to be transformed to MNI coordinates. The GM data were aligned and modulated for the preservation of GMV and smoothed with an 8 mm full width at half-maximum Gaussian kernel, using SPM12 [37]. After the normalized modulation, the resulting modulated images were preserved for the total amount of the GM signal, and the total TIV was applied as a correction measure.

### 2.7. Statistical Analysis

#### 2.7.1. Demographic and Clinical Data Analyses

The differences between groups in the demographic, normalization measure, and clinical data were conducted through a chi-square test for discrete variables (i.e., sex) and two-sample t-tests for continuous variables (i.e., age, total intracranial volume, TIV, SR), using IBM SPSS Statistics 25. The TIV was calculated as the sum of GMVs, white matter, and cerebrospinal fluid.

#### 2.7.2. VBM Analyses

The between-group differences in GMV were examined using VBM [38]. Whole-brain voxel-wise comparisons of GMV between groups were performed using two-sample t-tests to compare participants with and without phobia, using age, sex, and TIV as covariates in CAT12. The Gaussian random field theory [39,40] was performed to control for multiple comparisons with a significance threshold of a voxel-wise value of *p* < 0.001 and cluster probability of *p* < 0.05 [41]. In addition, all reported results were corrected for multiple comparisons using the false discovery rate (FDR). To assess the differences in total GMV between both groups with and without phobia, an ANOVA was used. Pearson correlation coefficients were calculated to evaluate the relationship between total GMV and SR scores. Nevertheless, an ANCOVA was applied to contrast the GMV between both phobic and non-fearful control groups, and to control for SR scores and age.

## 3. Results

### 3.1. Demographic, Normalization, and Clinical Characteristics

Significant differences between with and without phobia groups appeared in terms of age (group with phobia: mean = 35.16, SD = 11.11; group without phobia: mean = 22, SD = 5.09, t (60) = 6, *p* < 0.001) and anxiety symptoms (group with phobia: mean = 38.65, SD = 6.94; group without phobia: mean = 4.45, SD = 0.80, t (60) = 21.63, *p* < 0.001). The participants with phobia had significantly higher scores for anxiety symptoms and were older than those without phobia. Moreover, there were no significant differences in TIV—the normalization measure—between the two groups with and without phobia (group with phobia mean = 1466.29 cm^3^, SD = 26.27 cm^3^; group without phobia mean = 1515.39 cm^3^, SD = 154.41 cm^3^; t (60) = −1.27, *p* = 0.209). However, differences were found between males and females in TIV (F_1,60_ = 19.843, *p* < 0.001, mean males = 1636.23 cm^3^, SD = 135.789 cm^3^; mean females = 1452.61 cm^3^, SD = 131.195 cm^3^).

### 3.2. Groups Differences in GMV

**Figure 1:**
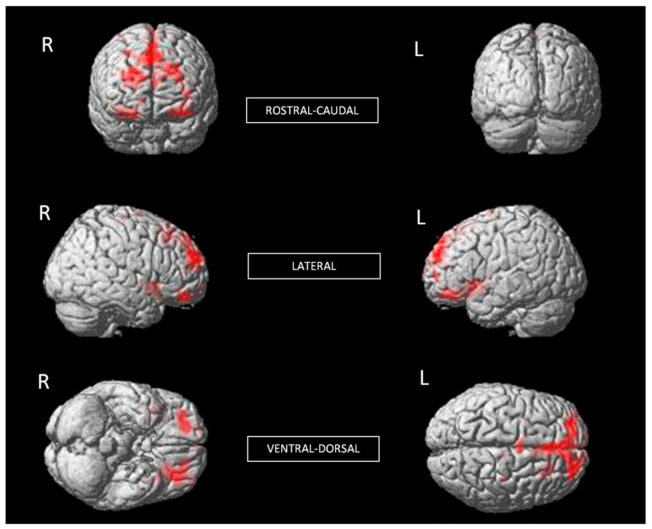
Significant differences in GMV of brain regions between non-fearful controls (larger) and individuals with phobia (corrected with Gaussian random field theory with a significance threshold of a voxel-wise value of *p* < 0.001 and cluster probability of *p* < 0.05).

Significant differences between with and without phobia groups appeared in total GMV (group with phobia: mean = 673,26 cm^3^, SD = 70.85 cm^3^; group without phobia: mean = 718.81 cm^3^, SD = 62.49 cm^3^, F_1,60_ = 7.20, *p* = 0.009). Total GMV correlated negatively with SR scores (r = −0.384, *p* = 0.002). ANCOVA revealed no significant effects of SR or age and did not exceed the cut-off point for considering them statistically significant (F_1,60_ = 33.56, *p* < 0.05). The whole-brain voxel-wise analysis showed that, compared with individuals without phobia, individuals with phobia had significantly smaller GMV in the right insula (t (60) = 5.18, *p* < 0.001); a cluster of right lateral, anterior, and inferior OFC (t (60) = 4.98, *p* < 0.001); a cluster of left posterior, medial, and lateral OFC, and left insula (t (60) = 4.86, *p* < 0.001); and a cluster of left superior medial frontal, right superior frontal, and right ACC (t (60) = 4.48, *p* < 0.001) (Table 1 and Figure 1). In all contrasts, the effect size was moderate.

**Figure 2:**
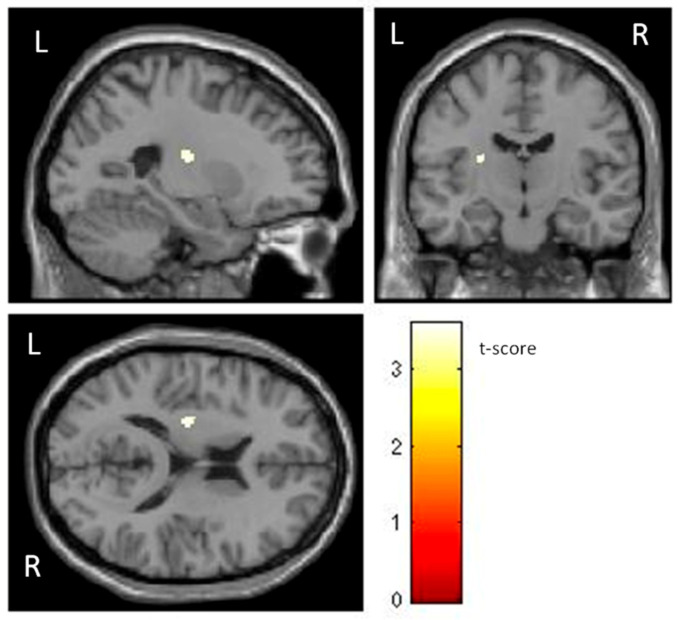
Significant differences in GMV of the putamen between individuals with phobia (larger) and non-fearful controls (corrected with Gaussian random field theory with a significance threshold of a voxel-wise value of *p* < 0.001 and cluster probability of *p* < 0.05). T-score is represented on a color bar.

Additionally, individuals with phobia showed a larger GMV in the left putamen than non-fearful controls (t (60) = 3.48, *p* < 0.001) (Table 1 and Figure 2), with a moderate effect size. The putamen is related to complex motor regulation and the facilitation of different types of learning.

## 4. Discussion

The aim of this study was to analyze whether certain areas responsible for acquiring and maintaining fear were structurally different between individuals with small animal specific phobia and non-fearful controls. The morphometric differences could suggest a greater vulnerability of phobic individuals to acquiring non-adaptive conditioned responses. Accordingly, individuals with small animal specific phobia showed smaller GMVs in cortical regions, such as the OFC and medial frontal cortex, and greater GMVs in the putamen than participants without phobia. The superior/medial frontal areas and ACC are involved in the elaboration of strategies for problem-solving and emotional regulation, while the OFC exerts cognitive control by reducing automatic responses from the amygdala, and the insula integrates interoceptive information.

Morphometric analysis revealed that a cluster of superior/medial frontal areas and ACC were diminished in phobic participants. In the same vein, decreased volumes in the lateral/medial PFC cortex have been found in GAD and panic disorder [19,26]. However, an increased volume in the left PFC, OFC, and ACC has been found when individuals with phobia were compared with non-fearful controls [30]. The superior/medial frontal areas and ACC are involved in planning and creating strategies for problem-solving and emotional regulation [42]. Therefore, decreased volume in these areas could be interpreted as an alteration to emotional regulation strategy planning to reduce fear in the face of the negative impact of phobias. These results should be interpreted with caution, because individuals with phobia could have a smaller innate GMV in these areas and therefore experience fear when confronted with a phobic stimulus (cause), or the continuous avoidance of such phobic stimuli could potentially lead to brain structure changes (consequence).

Additionally, our results showed smaller OFC and insula volumes in individuals with phobias compared with non-fearful controls, which is consistent with previous studies [19]. An increased GMV of the OFC has been associated with a high capacity for emotional self-regulation [43]. The OFC is responsible for top-down processes, exerting cognitive control by reducing automatic responses from the amygdala [44,45]. In young adults with social anxiety, an altered amygdala–orbitofrontal functional connectivity explains the effort of the PFC to control amygdala overactivity [46]. On the other hand, according to the fear network model, the insula integrates interoceptive information [47]. Taken together, the findings indicate that individuals with phobia would have difficulty integrating external information that would enhance learning new behaviors and would be carried away by the automatic triggers of emotion. Recent research suggests that the salience network and inhibitory control are altered in ADs [48]. Thus, individuals with phobia assign greater importance to the emotion generated by the feared stimulus than the regulatory mechanisms. Indeed, interventions based on exposure to the feared stimulus support the functional importance of the PFC which could suggest an attempt at emotional regulation or even the activity of other cognitive processes involved [49]. Therefore, phobias could be better explained by a model focusing on the control exerted by frontal areas as opposed to the dual model that emphasizes the effect of the deactivation of limbic areas.

However, in our study we found no significant GMV differences in amygdala. In this line, a meta-analysis reported no variations in the volume of the amygdala of individuals with SAD compared with healthy controls [22]. However, it did indicate diminished amygdala GMV in patients with spider phobia associated with higher anxiety symptom severity [28] and in GAD patients [50]. Treatment studies applying cognitive behavior therapy for SAD patients revealed a reduced GMV in amygdala and PFC, parietal–occipital regions after intervention [51]. Although functional studies found that interventions with exposure to real and virtual phobic stimuli were effective in decreasing anxiety responses and brain prefrontal activity after treatment, the amygdala remained activated [52].

Furthermore, the most striking finding of this study was a larger left putamen GMV that might be specific to phobias. This structure is related to complex motor regulation and the facilitation of different types of learning, namely, the operational learning associated with avoidance behavior [53]. Although most studies indicate a smaller putamen in ADs [20,22], a larger putamen volume has been identified in GAD [54] and SAD [27] compared with healthy controls. An increased putamen volume has also been found in individuals with anxious vulnerability [19,55]. During structural maturation in the brain, larger GMVs might reflect a lack of synaptic pruning and myelination, which could interfere with the efficiency of the corresponding psychological processes involved in this brain area [56]. Taken together, these results suggest that a pre-existing altered volume in certain brain areas could link learning impairment to anxious-fearful stimuli. Some authors suggest that atrophy in PFC areas could be a biomarker of vulnerability for the occurrence of ADs. The smaller size of these areas could interfere with emotional processing through top-down executive control mechanisms maintaining fear in the presence of the phobic stimulus [24]. Therefore, neurodevelopmental vulnerabilities would lead to the emergence of the disorder in the year prior to adolescence [7]. On the other hand, the increased putamen volume may be due to increased reactivity to threat cues or resistance to extinction. A possible explanation is that lengthy reinforcement of the behavior of escape from phobic stimulus would lead to a larger putamen volume [57]. In small animal phobia, cognitive behavioral therapy has shown efficacy in changing the emotional response, resulting in decreased severity of anxiety, as well as a reduced volume in brain areas, such as the supplementary motor area and PFC [31,52].

### 4.1. Limitations and Future Directions

This study has several limitations. First, the sample is small, and male individuals are underrepresented. In any case, the prevalence of phobias is more than double in females than in males [58]. Moreover, previous studies on GAD found no gender differences in GMVs [54]. Second, although both participant groups differed in age, most participants were over 20 years old. Hence brain maturity had already been reached. The GM volume increases until the age of four to six years, with development peaking at four years, whereas white matter increases until the age of 20 [59]. In the same line, Lebel et al. [60] found that deep GM structures, such as the putamen, thalamus, caudate nucleus, and globus pallidus reach maturity between 21 and 24 years. Although cortical and subcortical volume has also been found to decrease with age after the peaks [61]. However, in our study the total age span of both groups was similar so it is unlikely that a decrease in GM volume could occur due to the time course. In addition, the decrease in GMV was observed in specific areas linked to emotional regulation and appeared in the control group which was the youngest. Third, this study has not distinguished between different subtypes of animal phobias or recorded the possible comorbidity with other ADs. The results should therefore be taken with caution. Structural alterations may reflect neuroanatomical variations between different types of ADs [19]. Fourth, since the study is cross-sectional, the structural differences between individuals with phobia and non-fearful controls would be due to the pathogenesis of the disorder, a consequence of the disorder or other uncontrolled factors. Future research is needed to identify the structural changes and functional connectivity in order to establish a model that will explain both the shared elements and those specific to each type of phobia. It would be interesting to develop more precise techniques to identify structural variations in cortical and subcortical areas in fear-related disorders.

### 4.2. Conclusions

In sum, from our knowledge, this study is the first to examine the volumetric differences between participants with small animal phobia and non-fearful controls. The findings support the importance of the PFC regulatory role and the insula to a greater extent than limbic system deactivation. In other words, non-fearful controls showed a larger cortical GMV in areas associated with emotional regulation than individuals with phobia, although the subcortical areas linked to the limbic system showed no differences between either group (except for small differences in the putamen). Our findings have theoretical implications which contribute to a better understanding of the mechanisms underlying phobias that emphasize the predominant role of the PFC and the insula in fear regulation, both of which are diminished in individuals with phobia. On the other hand, brain areas linked to operational learning barely showed volumetric differences. These results would imply that specific phobia acquisition depends more on emotional regulatory processes than an alteration in areas associated with non-adaptive learning processes. The practical implications of these findings could be to improve the development of neuroimaging-based diagnostic markers to support clinical decisions, thereby improving diagnosis and prognosis. The prediction of an adaptive clinical response will depend on the different neural mechanisms involved in the conscious experience of fear, as well as in the defensive reaction to threat.

## Figures and Tables

**Table 1:** GMV differences between individuals with phobia and non-fearful controls.

Brain Structures	MNI Coordinates	k	t	*p*	r^2^
	x y z				
*Non-fearful controls > Individuals with phobia*					
R Insula	45 9 −9	961	5.18	<0.001	0.31
R Lateral OFC R Anterior OFC R Inferior OFC	24 48 −21	948	4.98	<0.001	0.29
L Posterior OFC L Middle OFC L Lateral OFC L Insula	−19 27 −13	2924	4.86	<0.001	0.28
L Superior Medial Frontal R ACC R Superior Frontal	−25 51 28	7104	4.48	<0.001	0.25
*Individuals with phobia > Non-fearful controls*					
L Putamen	−26 −15 15	60	3.58	<0.001	0.18

Note: L = Left, R = Right, OFC = Orbitofrontal cortex, ACC = Anterior cingulate cortex, k = Cluster size, t = Peak of t score, *p* = Probability for peak voxel (FDR corr.), r^2^ = effect size.

## Data Availability

The data presented in this study are available in the Supplementary Materials.

## Supplementary Materials

The following supporting information can be downloaded at: https://drive.google.com/drive/folders/1wVOdPCafjJWesA_iqVgl84rffCCfrWad?usp=sharing (accessed on 25 July 2022).
